# A Positive Semidefinite Safe Approximation of Multivariate Distributionally Robust Constraints Determined by Simple Functions

**DOI:** 10.1007/s10957-025-02791-5

**Published:** 2025-09-02

**Authors:** Jana Dienstbier, Frauke Liers, Jan Rolfes

**Affiliations:** 1 Department of Mathematics, Linköping University, SE-581 83 Linköping, Sweden; 2 Friedrich-Alexander-Universität Erlangen-Nürnberg, Cauerstr. 11, 91058 Erlangen, Germany

**Keywords:** Distributionally robust optimization, Robust optimization, Stochastic optimization, Mixed-integer optimization, 90C11, 90C17, 90C22, 90C34

## Abstract

Single-level reformulations of (nonconvex) distributionally robust optimization (DRO) problems are often intractable, as they contain semi-infinite dual constraints. Based on such a semi-infinite reformulation, we present a safe approximation that allows for the computation of feasible solutions for DROs that depend on nonconvex multivariate simple functions. Moreover, the approximation allows to address ambiguity sets that can incorporate information on moments as well as confidence sets. The typical strong assumptions on the structure of the underlying constraints, such as convexity in the decisions or concavity in the uncertainty found in the literature were, at least in part, recently overcome in [16]. We start from the duality-based reformulation approach in [16] that can be applied for DRO constraints based on simple functions that are univariate in the uncertainty parameters. We significantly extend their approach to multivariate simple functions, which leads to a considerably wider applicability of the proposed reformulation approach. In order to achieve algorithmic tractability, the presented safe approximation is then realized by a discretized counterpart for the semi-infinite dual constraints. The approximation leads to a computationally tractable mixed-integer positive semidefinite problem for which state-of-the-art software implementations are readily available. The tractable safe approximation provides sufficient conditions for distributional robustness of the original problem, i.e., obtained solutions are provably robust.

## Introduction

In this work, we consider distributionally robust optimization (DRO) models that are governed by multivariate simple functions that appear in many relevant contexts. Despite their nonconvexity, we aim for algorithmically tractable approximations that are based on duality arguments. The resulting solutions yield a safe approximation, which means that they are guaranteed to be robust for the original constraints.

The approach presented here starts from the considerations in [16] for constraints that are univariate in the uncertain parameters and generalizes the approach to the considerably more general case of constraints that are multivariate in the uncertainty. In the latter approach, a safe approximation was developed that leads to a mixed-integer linear optimization problem. Despite the NP-hardness of the latter, practically efficient algorithms and software are readily available. In addition, it could be proven that the safe approximation is asymptotically correct, i.e., it does not only yield robust solutions but asymptotically solves the original distributionally robust problem. In our generalization to the multivariate setting, we use the same notation as in [16]. For completeness of the exposition, we repeat the necessary ingredients. Let $x\in \mathbbm {R}^n$ denote the decision variables, $b\in \mathbbm {R}$ a scalar, $\mathcal {P}$ a set of probability measures on the compact domain $T\subseteq \mathbbm {R}^m$. We then model the uncertainty in our DRO with a random vector $t\in T$ distributed according to an (uncertain) probability measure $\mathbbm {P}\in \mathcal {P}$.

As typical in (distributionally) robust optimization, the task consists in determining decisions *x* that are feasible even in case the uncertain probability measures are chosen in an adversarial way which coined the name ’adversary’. In addition, in the case of an optimization model, the chosen robust solution shall lead to a best possible guaranteed objective value. Here, $v:\mathbbm {R}^n\times T\rightarrow \mathbbm {R}$ denotes a (possibly nonconvex) function that connects the decision variables *x* with the random vector *t*. Then, a *distributionally robust constraint* or DRO constraint is defined by

1 $$\begin{aligned} b\le \mathop {\textrm{min}}\limits _{\mathbbm {P}\in \mathcal {P}} \mathbbm {E}_{\mathbbm {P}}\left( v(x,t)\right) . \end{aligned}$$

Constraints of this form contain both the purely stochastic as well as the robust models as special cases. Indeed, setting $\mathcal {P}=\{\mathbbm {P}\}$, leads to a *stochastic constraint*:

$$\begin{aligned} b\le \mathbbm {E}_{\mathbbm {P}}\left( v(x,t)\right) . \end{aligned}$$

Stochastic optimization has been established for situations when uncertainty is distributed by some (known) distribution or when constraints must be met with a certain probability. It hedges against uncertainty in a probabilistic sense and implicitly assumes that the underlying distributions can be closely approximated or are even known exactly. We refer to [11] for a gentle introduction to stochastic optimization and to the surveys [33] and [35] particularly for discrete random variables.

Setting $\mathcal {P}=\{\delta _t: t\in T\}$, where $\delta _t$ denotes the Dirac point measures at $t\in T$, (1) yields a *robust constraint*

$$\begin{aligned} b\le \mathop {\textrm{min}}\limits _{t\in T} v(x,t). \end{aligned}$$

While more details on related literature will be given in a later section, we mention here the introductory textbooks for continuous robust optimization [6, 10] as well as combinatorial robust optimization  [20, 22].

In stochastic optimization, a large variety of efficient and elegant models and solution approaches have been established. However, in applications the underlying distributions are often unknown, which may result in low-quality or even infeasible results in case the underlying assumptions on the distributions are not satisfied.

In contrast, robust optimization offers a natural alternative whereby uncertainty sets are established a priori. Feasibility of an obtained solution is guaranteed for all possible outcomes of uncertainty within the uncertainty sets. A solution with the best guaranteed value is determined. Modelling and algorithmical aproaches consist in (duality-based) reformulations of the semi-infinite or exponentially large robust counterparts, if it is allowed by underlying structural assumptions such as convexity or, more generally, some underlying duality theory. If this is not possible, then decomposition algorithms are developed, possibly together with some approximation approaches if still the underlying robust problems are too demanding to solve.

In this work, we focus on distributional robust optimization (DRO). DRO determines robust solutions that are protected against uncertainty in the underlying distributions. These distributions are assumed to reside in a so-called ambiguity set of probability measures, denoted by $\mathcal {P}$ above. For distributionally robust optimization, we refer to the detailed surveys [34] and [26] as well as the references therein.

Generalizing from [16], we here allow the presence of multivariate simple functions *v*, i.e. $\text {dim}(T)=m>1$. The latter are basic building blocks in Lebesgue integrals. As simple functions are nonconvex, we cannot expect to derive an equivalent reformulation of the DRO model. However, our main contribution lies in the derivation of a mixed-integer positive semidefinite safe approximation, i.e., all obtained solutions are guaranteed to be robust. Due to the availability of state-of-the-art software implementations for mixed-integer positive semidefinite optimization, this proves the computational tractability of our modelling approach.

This work is structured as follows. Section 3 introduces the distributionally robust model, including simple functions, together with motivation and illustrative examples. Subsequently, Section 4 presents a new semi-infinite inner approximation of the robust counterpart, along with a suitable discretization. The result consists of a novel finite-dimensional mixed-integer positive semidefinite optimization model. The main contribution consists in showing that its feasible solutions are also feasible for the original robust DRO model.

## Literature Review

Next, we briefly review some relevant literature in optimization under uncertainty and distributional robustness in particular. Next to the textbooks [6, 10, 20, 22] mentioned above, relevant literature on robust optimization starts from the first treatments of linear optimization with uncertain coefficients [38] to a systematic study of linear optimization under uncertainty in e.g., [4] and [5]. In these approaches, duality-based reformulations have been developed that lead to algorithmically tractable robust counterparts for which practically usable solution approaches exist or software packages are available.

In order to push duality-based reformulation approaches even beyond linear and convex optimization, a wide variety of reformulations have been presented in [7]. If an underlying duality theory cannot be assumed, often decomposition approaches are developed. This is in particular the case for nonconvex robust optimization, where the optimization problem is nonconvex in the uncertainty. A practically efficient solution framework is given by an adaptive bundle approach [23], which has been integrated in an outer approximation procedure in [24] for additional discrete decisions. We refer to the survey [25] for additional references for nonlinear robustness.

Robust and stochastic constraints can be integrated either via so-called ’probust functions’, see e.g., [1] or [8] or via distributional robustness (DRO) from formula (1). Such integrated robust-probabilistic models contain advantages of both worlds, namely full protection as in the robust world together with limited prize of uncertainty protection as in stochastic optimization.

DRO surveys are presented in [34] and [26].

It is widely accepted that the right choice of ambiguity set is crucial both with respect to algorithmic tractability of the resulting robust counterparts as well as with respect to the obtained solution quality. Indeed, the ambiguity sets shall be chosen so that the most relevant uncertainties are considered while taking available partial information into account, but simultaneously overconservative solutions are avoided.

Discrepancy-based ambiguity sets assume a nominal, ’typical’ distribution and include distributions within a certain distance of it, where the Wasserstein metric is a natural distance [17]. Ambiguity sets have, for example, also been derived from phi-divergence [21], from likelihood ambiguity sets in [39], as well as from statistical hypothesis tests [9].

Going beyond convex models, in our approach we allow the presence of nonconvex simple functions and mainly focus on moment-based ambiguity sets. The moments of distributions are uncertain but are assumed to satisfy predetermined bounds. For convex models, mean-variance or value-at-risk measures are studied in [19], whereas moment information is used in [32]. The article [41] uses Slater conditions to show the correctness of a duality-based reformulation of the robust counterpart, together with discretization schemes to determine approximate solutions. [14] presents exact reformulations of convex DRO problems, where confidence regions of some moments are considered.

For convex models, some recent works combine partial information based on discrepancies as well as on moments of the distribution to define ’tight’ ambiguity sets. In this flavor, in [13], the authors derive efficient inner and outer approximations for DRO where both moment as well as Wasserstein ambiguity sets can be used simultaneously.

One of the challenges of incorporating additional information into moment-based ambiguity sets is addressed by the authors of [31], who provide a positive semidefinite (SDP) reformulation of (1) for cases where the probability distribution is known to be unimodal and the moments are fixed. On the other hand, [40] presents a duality-based reformulation of (1) that incorporates information on the confidence sets and assumes convex optimization problems. Under these assumptions, the approach can be applied to a DRO with (1) as a constraint.

The recent work [28] also allows hybrid ambiguity sets by enriching Wasserstein balls with additional moment information. For discrete decisions, approximations are presented.

In [3], the authors go a step further and consider multi-stage DRO settings. Via scenario grouping, they present bounds taking conditional ambiguity sets into account that occur in the multi-stage mixed-integer DRO setting.

In our work, we use moment-based ambiguity sets similar to [14], which consider mean and covariance matrix ranges along with confidence set information as in [40]. Our main contribution is to allow the presence of nonconvexities, which considerably extends existing reformulation approaches. Indeed, in addition to being able to model tight ambiguity sets, we also allow that the optimization models contain multivariate nonconvex simple functions. These functions can approximate any, even nonconvex, continuous function.

Due to these nonconvexities, standard reformulation approaches based on duality cannot be applied. In order to apply them nevertheless, we first approximate the nonconvexities appropriately by convex functions for which we then present reformulations to optimization problems in function space. [16] considers the univariate case and presents a safe approximation that is based on mixed-integer linear constraints. In addition, they could prove that the safe approximation converges to the true robust counterpart solution, rendering the approximation asymptotically a correct equivalent reformulation. For the multivariate setting considered here, appropriate discretizations result in mixed-integer positive-semidefinite optimization problems. The latter are algorithmically tractable and can be solved via available software. As a result, we present reformulation approaches for such nonconvex multivariate DRO problems that allow algorithmically tractable solution of the resulting robust counterparts.

## Problem Setting and Notation

We stick to the notation from [16] and summarize the main modelling here for completeness of our exposition.

### DRO Constraints Containing Simple Functions

The DRO constraints considered in the present article are defined by functions *v*(*x*, *t*) that consist of multivariate *simple functions*, i.e., finite linear combinations of indicator functions:

$$\begin{aligned} v(x,t)=\sum _{i=1}^k x_i \mathbbm {1}_{X_i}(t), \text { where } \mathbbm {1}_{X_i}(t):={\left\{ \begin{array}{ll} 1 &  \text { if } t\in X_i\\ 0 &  \text { otherwise.} \end{array}\right. } \end{aligned}$$

The functions of type $\mathbbm {1}_{X_i}$ are denoted as *indicator functions* as they indicate whether $t\in X_i$ holds or not. The sets $X_i$ can be considered as events in the probability space given by $\mathbbm {P}$. In fact, considering functions *v* as above in (1) leads to

$$\begin{aligned} \mathbbm {E}_{\mathbbm {P}}(v(x,t)) = \mathbbm {E}_{\mathbbm {P}}\left( \sum _{i=1}^k x_i\mathbbm {1}_{X_i}(t)\right) = \sum _{i=1}^k x_i \mathbbm {P}(X_i) \end{aligned}$$

and consequently the following formulation of (1):

2 $$\begin{aligned} b \le \mathop {\textrm{min}}\limits _{\mathbbm {P}\in \mathcal {P}} \sum _{i=1}^k x_i \mathbbm {P}(X_i). \end{aligned}$$

We note that one may see (2) as a robust chance constraint that is allowed to consist of simple functions. The decisions may either influence the height $x_i$ of an indicator function, see Case 1, or will determine the underlying domains $X_i$, see Case 2. In the remainder of this paper, we will investigate both situations separately to ease the presentation. However, the safe approximation presented in Theorem 4.3 can be extended to incorporate both cases simultaneously.

**Case 1:** Suppose that the sets $X_i\subseteq \mathbbm {R}^m$ are given; then we ask for optimal decisions $x_i$ such that the DRO constraint (1) is satisfied.

3a $$\begin{aligned} \max _{x\in C}\  &c(x): \end{aligned}$$

3b $$\begin{aligned} \text {s.t.}\,&b \le \mathop {\textrm{min}}\limits _{\mathbbm {P}\in \mathcal {P}} \sum _{i=1}^k x_i \mathbbm {P}(X_i), \end{aligned}$$

where $c:\mathbbm {R}^n\rightarrow \mathbbm {R}$ denotes a concave objective function, $C\subseteq \mathbbm {R}^n$ denotes a set of additional convex constraints. Note that in Case 1, we have that $n=k$.

We demonstrate the generality of (3) by an academic example on the mean-variance model from portfolio optimization; see Example 3 in [36]: To this end, suppose one aims to minimize the risk of a portfolio. Moreover, one only has *n* risky assets $A_i$ available. Let these assets provide a revenue $r_i$ in case of an event $X_i$ and 0 otherwise, i.e. $A_i=r_i \mathbbm {1}_{X_i}$ and let the $A_i$ be independently, identically distributed with probability $\mathbbm {P}\in \mathcal {P}$, where $\mathcal {P}$ denotes a pre-defined ambiguity set as described in Section 1. Assume that the covariance matrix of the assets $A_i$ is dominated by a matrix $\varSigma$, i.e., $0\preceq \text {Var}(A)\preceq \varSigma$ and we ask for a guaranteed revenue *w* of our portfolio. Then, the mean-variance model reads:

$$\mathop {\textrm{min}}\limits _x x^\top \varSigma x: \mathop {\textrm{min}}\limits _{\mathbbm {P}\in \mathcal {P}} \mathbbm {E}_{\mathbbm {P}}\left( \sum _{i=1}^n x_iA_i\right) \ge w, \sum _{i=1}^n x_i=1, x\ge 0,$$

which for i.i.d. assets $A_i$ is equivalent to

$$\begin{aligned} -\max _x -\sum _{i=1}^n \sigma _i x_i^2:\ \mathop {\textrm{min}}\limits _{\mathbbm {P}\in \mathcal {P}} \sum _{i=1}^n x_ir_i\mathbbm {P}(X_i) \ge w, \sum _{i=1}^n x_i=1, x\ge 0. \end{aligned}$$

This is indeed a special case of (3) since nonnegative $\sigma _i,x_i$ lead to a concave objective function and $\left\{ x\in \mathbbm {R}^n: \sum _{i=1}^n x_i=1, x\ge 0\right\}$ denotes a convex set for which, e.g., the methods from [40] can be applied. Thus, although addressing Case 1 as well, in the present article we focus on the following case, where we consider the sets $X_i$ as decision variables.

**Case 2:** Suppose the coefficients $x_i$ are given parameters and the sets $X_i=[x_i^-,x_i^+]\subseteq \mathbbm {R}^m$ determine hypercubes. Consider the boundaries of these hypercubes as decision variables. In addition, we assume w.l.o.g. that $X_i\subseteq T$ for well-posedness of $\mathbbm {P}(X_i)$ and additionally assume a linear objective function for ease of presentation. In particular, we consider:

4a $$\begin{aligned} \max _{((x^-)^\top ,(x^+)^\top )\in C}\  &\sum _{i=1}^k\sum _{j=1}^m c^-_{ij}x^-_{ij}+c^+_{ij}x^+_{ij}: \end{aligned}$$

4b $$\begin{aligned} \text {s.t.}\  &b \le \mathop {\textrm{min}}\limits _{\mathbbm {P}\in \mathcal {P}} \sum _{i=1}^k x_i \mathbbm {P}([x_i^-,x_i^+]), \end{aligned}$$

where $C\subseteq \mathbbm {R}^{2km}$ denotes a polytope of $n=2k$ decision vectors, each of dimension *m*. Note, that Case 2 appears to be more challenging than Case 1 as the function

$$v(x^-,x^+,t):=\sum _{i=1}^k x_i\mathbbm {1}_{[x_i^-,x_i^+]}(t)$$

is not only nonconvex in *t* but also in $((x_i^-)^\top ,(x_i^+)^\top )$. Despite this mathematical challenge, this case already covers interesting applications in chemical separation processes, as is illustrated in [16].

Let us now introduce essential notation and concepts. We refer to [2] and [37] for more information. The main challenges in Problems (3) and (4) arise from the DRO constraints (3b) and (4b), since these constraints cannot be formulated with the canonic Euclidean inner product. Consequently, standard reformulation arguments from robust optimization, such as replacing the inner adversarial optimization problem by the feasible region of its dual and solving the resulting model as a standard finite-dimensional convex problem, do not apply. However, the following inner product, illustrated in Section III.3.2 in [2], allows a similar reformulation of (3b) and (4b):

Let $\mathbbm {P}$ denote a probability measure on the compact domain *T* that is defined by a probability density $\rho _{}({t})$, i.e., $d\mathbbm {P}= \rho _{}({t})dt.$ According to Riesz-Markov-Kakutani representation theorem $\mathbbm {P}$ is unique, i.e., it is the only solution that satisfies $I(f)=\int f d\mathbbm {P}$ for the linear functional $I:\mathcal {C}(T)\rightarrow \mathbbm {R}$ defined by $I(f):=\int _0^T f(t)\rho _{}({t})dt.$ The corresponding inner product

$$\langle f, \mathbbm {P}\rangle :=\int _T f d\mathbbm {P}$$

then constitutes a *duality*, i.e. a non-degenerate inner product. Moreover, this duality is more generally defined on *signed Radon measures*, denoted by $\mathcal {M}(T)$.

Suppose, we know a continuous approximation of the indicator function $\mathbbm {1}_{X_i}$, denoted by $\mathbbm {1}_{X_i}^c$. Then, we observe that the above product $\langle \cdot , \cdot \rangle : \mathcal {C}(T)\times \mathcal {M}(T)\rightarrow \mathbbm {R}$, enables us to approximate (2) via the function $\sum _{i=1}^k x_i\mathbbm {1}_{X_i}\in \mathcal {C}(T)$ and the probability measure $\mathbbm {P}\in \mathcal {M}(T)$ as follows:

5a $$\begin{aligned} b \le \mathop {\textrm{min}}\limits _{\mathbbm {P}}~&\langle \sum _{i=1}^k x_i\mathbbm {1}_{X_i}^c,\mathbbm {P}_{}\rangle&\end{aligned}$$

5b $$\begin{aligned} \text {s.t.}~&\mathbbm {P}_{} \in \mathcal {M}(T)_{\ge 0} \end{aligned}$$

5c $$\begin{aligned}&\langle 1, \mathbbm {P}_{} \rangle \ge 1 \end{aligned}$$

5d $$\begin{aligned}&\langle -1, \mathbbm {P}_{} \rangle \ge -1, \end{aligned}$$

where $\mathcal {M}(T)_{\ge 0}$ denotes the cone of nonnegative Radon measures. Furthermore, Constraints (5b) – (5d) require $\mathbbm {P}_{}$ to be a probability measure.

### Strengthening DRO Models by Moment Control and Confidence Sets

One of the major challenges in distributional robustness consists in choosing additional constraints for (5) is on the one hand, algorithmically tractable, but on the other hand, also large enough to protect the solutions *x* (in Case 1) and $x^-,x^+$ (in Case 2) against all *realistic* uncertainties. Moreover, one aims to avoid including unrealistic uncertainties as those render the decisions *x* and $x^-,x^+$ too conservative. Within our setting, it is also possible to add additional information on the uncertain probability distributions. This leads to additional constraints that can be added to (5) while maintaining algorithmic tractability.

First, we aim at bounding the *first moment*, i.e. the expectation $\mathbbm {E}_{\mathbbm {P}_{}}(t)$, of $\mathbbm {P}_{}$. The authors in [31] and other sources assume perfect knowledge about the first moment, whereas the authors of [14] only assume that the first moment is contained in an ellipsoid. In this article, we follow the latter modeling and assume that an estimate of the correct expectation $\mu _{}$ and covariance matrix $\varSigma$ is known. Moreover, we assume that the ellipsoidal uncertainty set containing $\mathbbm {E}_{\mathbbm {P}_{}}(t)$ is shaped by $\mu _{}$, $\varSigma$ and a third parameter $\varepsilon _{\mu _{}}>0$, that determines its size. The ellipsoidal uncertainty set is then given by

$$\varepsilon _{\mu  }-(\mathbbm {E}_{\mathbbm {P} }(t)-\mu  )^\top \varSigma (\mathbbm {E}_{\mathbbm {P} }(t)-\mu  )\ge 0, \varSigma \succeq 0.$$

In order to reformulate the above constraint by means of an inner product $\langle \cdot , \mathbbm {P}_{}\rangle$, we apply Schur’s complement and obtain the following equivalent SDP constraint, which fits the setting in (5):

6 $$\begin{aligned} \left\langle \begin{bmatrix} \varSigma &  t-\mu _{}\\ (t-\mu _{})^\top &  \varepsilon _{\mu _{}} \end{bmatrix}, \mathbbm {P}_{}\right\rangle \succeq 0. \end{aligned}$$

Similarly, one may assume that the underlying uncertain probability measure is given by a monomodal density function; see e.g., [31]. Computationally, this assumption has the advantage, that, if $\mathcal {P}$ contains monomodal distributions with fixed first and second moments, (5) can be reformulated as an SDP. This is one of the main results in [31]. However, the corresponding SDP is not easy to incorporate into either (3) or (4) as it generally leads to bilinear terms and thereby intractable counterparts for both (3) and (4). In particular, [34] state that “with the current state of literature, monomodality cannot be modeled in a tractable manner.” To circumvent this obstacle, we exploit the fact that monomodal distributions tend to have a relatively small variance. Thus, similar again to [14], in addition to the bounds on the first moment, we impose an upper bound on the *second moment* as follows

7 $$\begin{aligned} \langle -(t-\mu _{})(t-\mu _{})^\top ,\mathbbm {P}_{}\rangle \succeq -\varepsilon _\varSigma \varSigma \end{aligned}$$

or, equivalently $\text {Var}_{\mathbbm {P}_{}}(t)\preceq \varepsilon _\varSigma \varSigma$. Here, $\varepsilon _\varSigma \ge 1$ measures the maximum deviation of the covariance matrix compared to its estimate $\varSigma$.

Finally, we add *confidence set* constraints, see e.g., [40], where we restrict the probability of certain subsets $T_i\subseteq T$, i.e.,

8 $$\begin{aligned} \langle \text {sign}(\varepsilon _i)\mathbbm {1}_{T_i}^c(t), \mathbbm {P}_{} \rangle \ge \varepsilon _i \text { for every } i\in I. \end{aligned}$$

Note that these constraints give us a lot of modeling power as we can model $\mathbbm {P}_{}(T_i)\ge \varepsilon _i$ with $\varepsilon _i>0$ and $\mathbbm {P}_{}(T_i)\le -\varepsilon _i$ with $\varepsilon _i<0$. In particular, the normalization constraints (5c) and (5d) fall in this framework and will be modeled by setting $T_1=T_2=T$ and $\varepsilon _1 = -1, \varepsilon _2=1$ throughout the remainder of this article.

### Relation to the Literature

In the existing literature, distributionally robust constraints are often encoded with the expectation $\mathbbm {E}_{\mathbbm {P}}(v(x,t))$, which in the present paper encodes the expectation of a nonconvex, in our case a piecewise-constant, function *v* in *t* by $\mathbbm {E}_{\mathbbm {P}}(v(x,t)) = \sum _{i=1}^k x_i \mathbbm {P}(X_i)$. Dropping the convexity assumption poses a stark contrast to the results in [40] and [14], where the underlying function *v*(*x*, *t*) has to be both convex and piecewise-affine in *x* and *t*, see Condition (C3) in [40] and Assumption 2 in [14]. However, [40] and [41] present exceptions to these assumptions for specific cases, namely a very low number |*I*| of confidence sets, see Observation 1ff in the electronic compendium of [40] or even $|I|=0$ ( [41]). As we consider indicator functions $\mathbbm {1}_{X_i}(t)$, that generally do not satisfy any of those assumptions, we attempt to extend the existing literature to nonconvex functions *v*. Moreover, in contrast to [16], we allow *T* to be multivariate and consider simple functions $\sum _{i=1}^k x_i\mathbbm {1}_{[x_i^-,x_i^+]}(t)$ instead of either sole indicator functions with $k=1$ or simple functions with the simplifying assumption, that the *m* entries of *t* are independent. This increased generality is achieved at the cost of a potentially worse approximation accuracy.

Lastly, we briefly mention the differences of our approach to discrepancy-based DRO models that require an estimator for the true probability distribution $\hat{\rho }$ and restrict $\mathcal {P}$ based on a given metric, e.g., the Wasserstein metric. Here, given an estimated $\hat{\rho }$, these ambiguity sets are formed of all probability distributions, that originate from $\hat{\rho }$ by transferring at most a given probability mass. We refer to the excellent review [34] for further details.

## Distributionally Robust Constraints Dependent on Simple Functions

For both Cases 1 and 2 from Section 3, we consider the DRO constraint (5) where $\mathcal {P}$ is defined by (6), (7) and (8). To this end, let again $b\in \mathbbm {R}$, $T\subseteq \mathbbm {R}^m$ be a compact set, and $I\subseteq \mathbbm {N}$ denote a finite index set. Next we define the considered ambiguity set. We assume a ’typical’, i.e., nominal distribution with mean $\mu _{} \in \mathbbm {R}^m$ and covariance matrix $\varSigma \in \mathbbm {R}^{m\times m}$ is given, for example, from expert knowledge or by estimation from given data. In formulas, we consider

9a $$\begin{aligned} b \le \mathop {\textrm{min}}\limits _{\mathbbm {P}_{}}~&\langle \sum _{i=1}^k x_i \mathbbm {1}_{X_i}^c,\mathbbm {P}_{}\rangle  &   \end{aligned}$$

9b $$\begin{aligned} \text {s.t.}~&\mathbbm {P}_{} \in \mathcal {M}(T)_{\ge 0} \end{aligned}$$

9c $$\begin{aligned}&\langle \begin{bmatrix} \varSigma &  t-\mu \\ (t-\mu _{})^\top &  \varepsilon _{\mu _{}} \end{bmatrix}, \mathbbm {P}_{}\rangle \succeq 0 \end{aligned}$$

9d $$\begin{aligned}&\langle -(t-\mu _{})(t-\mu _{})^\top ,\mathbbm {P}_{}\rangle \succeq -\varepsilon _\varSigma \varSigma  &   \end{aligned}$$

9e $$\begin{aligned}&\langle \text {sign}(\varepsilon _i)\mathbbm {1}_{T_i}^c(t), \mathbbm {P}_{} \rangle \ge \varepsilon _i  &   i\in I , \end{aligned}$$

where a choice of $T_1=T, \varepsilon _1=-1$ and $T_2=T, \varepsilon _2=1$ implies that $\mathbbm {P}_{}(T)=1$, i.e. $\mathbbm {P}_{}$ is a probability measure on *T*. In the following, we aim at deriving an algorithmically tractable reformulation of this set of constraints. We note that in order to dualize (9), we consider continuous approximators $x_i \mathbbm {1}_{X_i}^c, \text {sign}(\varepsilon _i)\mathbbm {1}_{T_i}^c$ of the indicator functions $x_i \mathbbm {1}_{X_i}, \text {sign}(\varepsilon _i)\mathbbm {1}_{T_i}$. The existence of approximators that are arbitrarily close to the indicator functions is given by the seminal Lemma of Urysohn, see e.g., [30]. In particular, we choose $\mathbbm {1}^c_{X_i} \ge \mathbbm {1}_{X_i}$, an upper approximator whenever $x_i\ge 0$ and a lower approximator whenever $x_i<0$. The opposite approximators are chosen for $\mathbbm {1}_{T_i}$, i.e., we choose $\mathbbm {1}_{T_i}^c\le \mathbbm {1}_{T_i}$ if $\varepsilon _i\ge 0$ and $\mathbbm {1}_{T_i}^c\ge \mathbbm {1}_{T_i}$ whenever $\varepsilon _i<0$. This establishes the following key property:

10 $$\begin{aligned} x_i\mathbbm {1}^c_{X_i} \ge x_i\mathbbm {1}_{X_i}\text { and } \text {sign}(\varepsilon _i)\mathbbm {1}^c_{T_i} \le \text {sign}(\varepsilon _i)\mathbbm {1}_{T_i}. \end{aligned}$$

In the following, we will define necessary ingredients for being able to reformulate such a DRO constraint by dualizing (9). Subsequently, a tractable and high-quality inner approximation of the resulting constraint will be obtained. We first employ duality theory using an adjoint operator:

### Remark 4.1

Let $\mathcal {S}^r$ denote the set of symmetric *r* by *r* matrices. It might not be immediately clear whether an adjoint operator with respect to the primal operator $\mathcal {A}: \mathcal {M}(T) \rightarrow \mathcal {S}^{m+1}\times \mathcal {S}^m \times \mathbbm {R}^I$ of (9) exists. However, it is constructed in a quite straightforward manner: First, we observe that for the inner products containing matrices $A\in \mathcal {S}^r$, we have

$$\langle \langle A, \mathbbm {P}  \rangle , Y \rangle _F = \langle \langle A, Y\rangle _F, \mathbbm {P}  \rangle \text { for arbitrary }\mathbbm {P} \in \mathcal {M}(T),Y\in \mathcal {S}^r,$$

where, $\langle \cdot ,\cdot \rangle _F: \mathcal {S}^r\times \mathcal {S}^r\rightarrow \mathbbm {R}$ denotes the Frobenius inner product. In particular, for $r\in \{m,m+1\}$, this includes the matrices

$$A\in \left\{ \begin{bmatrix} \varSigma &  t-\mu  \\ (t-\mu  )^\top &  \varepsilon _{\mu  } \end{bmatrix}, -(t-\mu  )(t-\mu  )^\top \right\} .$$

For the inner products containing only the entries $\text {sign}(\varepsilon _i) \mathbbm {1}_{T_i}^c$ of $\mathcal {A}$, we have

$$\langle \text {sign}(\varepsilon _i) \mathbbm {1}_{T_i}^c, \mathbbm {P}  \rangle y = \langle \text {sign}(\varepsilon _i) \mathbbm {1}_{T_i}^c y, \mathbbm {P}  \rangle \text { for every }\mathbbm {P} \in \mathcal {M}(T), y\in \mathbbm {R}.$$

Hence, we have constructed an adjoint operator $\mathcal {B}: \mathcal {S}^{m+1}\times \mathcal {S}^m\times \mathbbm {R}^I\rightarrow \mathcal {C}(T)$ to $\mathcal {A}$, which is defined by

$$\left\langle \begin{bmatrix} \varSigma &  t-\mu  \\ (t-\mu  )^\top &  \varepsilon _{\mu  } \end{bmatrix}, Y_1\right\rangle + \langle -(t-\mu  )(t-\mu  )^\top , Y_2\rangle + \sum _{i\in I}\text {sign}(\varepsilon _i) \mathbbm {1}_{T_i}^c y_i.$$

Moreover, $\mathcal {B}$ is unique due to Riesz’ representation theorem, see e.g., [12].

With this adjoint operator, we derive the following dual program for (9):

11a $$\begin{aligned} b \le \max _{y_i,Y_1,Y_2}~&\sum _{i\in I} \varepsilon _i y_i -\varepsilon _\varSigma \langle \varSigma , Y_2 \rangle \end{aligned}$$

11b $$\begin{aligned} \text {s.t.}~&\sum _{i=1}^k x_i \mathbbm {1}_{X_i}^c - \left\langle \begin{bmatrix} \varSigma &  t-\mu _{}\\ (t-\mu _{})^\top &  \varepsilon _{\mu _{}} \end{bmatrix} , Y_1 \right\rangle -\langle -(t-\mu _{})(t-\mu _{})^\top , Y_2\rangle \nonumber \\&-\sum _{i\in I} \text {sign}(\varepsilon _i) \mathbbm {1}_{T_i}^c y_i \in \mathcal {C}(T)_{\ge 0} \end{aligned}$$

11c $$\begin{aligned}&Y_1 \in \mathcal {S}^{m+1}_{\succeq 0}, Y_2 \in \mathcal {S}^m_{\succeq 0}, y \in \mathbbm {R}_{\ge 0}^I, \end{aligned}$$

where $\mathcal {C}(T)_{\ge 0}$ denotes the cone of the continuous, nonnegative functions on *T*.

As usual in reformulation approaches in robust optimization, we aim to apply strong duality. Indeed, next we establish strong duality between (9) and (11) that can be seen as a direct corollary of Corollary 3.0.2 in [37] or as a direct consequence of the dualization theory illustrated, e.g., in [2].

### Theorem 4.1

Suppose that $\mathbbm {P}_{} \sim \mathcal {N}(\mu _{},\varSigma )$ is both, a strictly positive Radon measure and feasible for (9). Then, the duality gap of the problems (9) and (11) is zero.

### Proof

We observe that $\mathbbm {P}_{} \sim \mathcal {N}(\mu _{},\varSigma )$ is feasible for (9), i.e. (9) is “consistent” in the definition of Shapiro. Furthermore, *T* is compact and the functions in the objective as well as in the constraints of (9) are continuous. Due to the isometry of the metric spaces $(\mathcal {S}^r, \langle \cdot , \cdot \rangle _F)$ and $(\mathbbm {R}^{\frac{r(r-1)}{2}}, \langle \cdot , \cdot \rangle )$, we further reformulate (9) as a conic program with $\mathcal {A}\mathbbm {P}_{} -b\in K$, where the cone $K\subseteq \mathbbm {R}^{(m+1)m/2 + m(m-1)/2+|I|}$. Hence, strong duality follows from Corollary 3.1 in [37]. $\square$

### Computation of Feasible Solutions by a Discretized Robust Counterpart

In this section, we derive an algorithmically tractable model for the robust counterpart (11). A standard approach to find an approximate solution to this semi-infinite (SIP) problem is to sample the semi-infinite constraint (11b) and solve the resulting finite-dimensional SDP that only contains the sampled constraints. However, a feasible solution to a finite subset of the constraints in (11b) does not necesarily satisfy (11b) itself. This means that the obtained solution may not satisfy (11) and thus by solving Case 1 or 2 with respect to this relaxation of (11), we might obtain a solution that is not necessarily protected against the uncertainties in the ambiguity set $\mathcal {P}$, i.e., is not robust and does not necessarily satisfy (9).

In this work, we however aim for a robust constraint for $\mathcal {P}$ as for many applications a guaranteed protection is important, e.g., in medical applications.

To this end, we propose a discretization scheme that provides an inner approximation of (11b). This means that every solution of the discretization of (11) will indeed satisfy (11) and thereby guarantee that the corresponding decision variables $x_i$ for Case 1 and $x_i^-,x_i^+$ for Case 2 are feasible for (9). This robust formulation will make use of Lipschitz continuity of the non-indicator functions in (11b), i.e., the Lipschitz continuity of the polynomial

$$p_Y(t):=\left\langle \begin{bmatrix} \varSigma &  t-\mu  \\ (t-\mu  )^\top &  \varepsilon _{\mu  } \end{bmatrix}, Y_1 \right\rangle +\langle (t-\mu  )(t-\mu  )^\top , Y_2\rangle .$$

In fact, the polynomial $p_Y$ is Lipschitz continuous since *T* is compact and its coefficients $Y_1,Y_2$ are bounded:

#### Lemma 4.1

Let $T_1=T$ and $\varepsilon _1=-1$. Furthermore, for every $i\in I\setminus \{1\}$ let $\mu _{} \in T_i$ if $\varepsilon _i>0$ and $\mu _{} \notin T_i$ if $\varepsilon _i<0$. Then, the polynomial $p_Y(t)$ is Lipschitz continuous in *t* with a uniform Lipschitz constant *L*.

#### Proof

Due to the compactness of *T*, it suffices to show that for every feasible solution of (11) the entries $Y_1,Y_2$ are bounded. To this end, let $\varepsilon _1=-1, \varepsilon _2=1$. In addition, we assume w.l.o.g. $\varepsilon _i >0$ for every $i\in I \setminus \{1\}$. This is due to the fact that every constraint

$$ \langle \text {sign}(\varepsilon _i)\mathbbm {1}_{T_i}^c,\mathbbm {P}\rangle \ge \varepsilon _i \text { with } -1 \le \varepsilon _i < 0$$

can equivalently be expressed by

$$\langle \mathbbm {1}_{T_i^C}^c,\mathbbm {P}\rangle \ge 1+\varepsilon _i.$$

In order to prove this equivalence, we note that $\text {sign}(\varepsilon _i)=-1$, add 1 on both sides and consider the complement $T_i^C$ of $T_i$.

Now, we first prove that $\text {Tr}(Y_1)<\infty$: Let $t=\mu _{}$, $v_i$ be the eigenvectors and $\lambda _i$ the eigenvalues of $Y_1$ then (11b) implies

12 $$\begin{aligned} \begin{aligned} \lambda _{\mathop {\textrm{min}}\limits }\left( \begin{bmatrix} \varSigma &  0 \\ 0 &  \varepsilon _{\mu _{}} \end{bmatrix}\right) \text {Tr}(Y_1)&= \sum _{i=1}^{m+1} \lambda _i \lambda _{\mathop {\textrm{min}}\limits }\left( \begin{bmatrix} \varSigma &  0 \\ 0 &  \varepsilon _{\mu _{}} \end{bmatrix}\right) \overset{*}{\le }\ \sum _{i=1}^{m+1} \lambda _i v_i^\top \begin{bmatrix} \varSigma &  0 \\ 0 &  \varepsilon _{\mu _{}} \end{bmatrix} v_i\\&\le \left\langle \begin{bmatrix} \varSigma &  0 \\ 0 &  \varepsilon _{\mu _{}} \end{bmatrix}, Y_1 \right\rangle \overset{{(11b)}}{\le } \sum _{i=1}^k x_i \mathbbm {1}_{X_i}^c(\mu _{}) - \sum _{i\in I} \text {sign}(\varepsilon _i) y_i, \end{aligned} \end{aligned}$$

where (*) holds due to the Rayleigh-Ritz principle; see e.g. [12] for further details. We show that (12) is bounded from above for every feasible solution to (11) by considering the following LP:

13 $$\begin{aligned} \mathop {\textrm{min}}\limits _{y\in \mathbbm {R}^I_{\ge 0}} \sum _{i\in I} \text {sign}(\varepsilon _i) \mathbbm {1}_{T_i}^c(\mu _{})y_i:\ \sum _{i\in I}\varepsilon _i y_i \ge b, \end{aligned}$$

whose constraint can be derived from (11a) and the fact that both $\varSigma$ and $Y_2$ are positive semidefinite. Moreover, this is equivalent to

$$\begin{aligned} \mathop {\textrm{min}}\limits _{y\in \mathbbm {R}^I_{\ge 0}} -y_1+\sum _{i\in I \setminus \{1\}} y_i:\ \sum _{i\in I}\varepsilon _i y_i \ge b. \end{aligned}$$

due to $\mu _{}\in T_i$ for every $i\in I$. Furthermore, it is bounded from below by 0 since its dual LP:

$$\begin{aligned} \max _{z\ge 0} b z : - z&\le -1,\\ \varepsilon _i z&\le 1  &   \text { for every } i\in I\setminus \{1\}, \end{aligned}$$

is feasible for $z=1$ since w.l.o.g. $|\varepsilon _i|\le 1$. Consequently, this provides a lower bound of *b* to (13) and thereby an upper bound to $\text {Tr}(Y_1)$ via (12).

Let $\lambda _{\mathop {\textrm{min}}\limits }(\varSigma )> 0$ denote the minimal eigenvalue of $\varSigma$ and $\lambda _i$ the eigenvalues of $Y_2$ with respect to eigenvector $v_i$. Then, on the one hand, we have

14 $$\begin{aligned} \begin{aligned} \varepsilon _\varSigma \lambda _{\mathop {\textrm{min}}\limits }(\varSigma ) \text {Tr}(Y_2)&= \varepsilon _\varSigma \sum _{i=1}^m \lambda _i \lambda _{\mathop {\textrm{min}}\limits }(\varSigma ) \overset{(*)}{\le }\ \varepsilon _\varSigma \sum _{i=1}^m \lambda _i v_i^\top \varSigma v_i \\  &= \varepsilon _\varSigma \left\langle \varSigma , \sum _{i=1}^m \lambda _i v_iv_i^\top \right\rangle = \varepsilon _\varSigma \langle \varSigma , Y_2 \rangle \overset{(11a)}{\le } \sum _{i\in I} \varepsilon _i y_i \end{aligned} \end{aligned}$$

where (*) holds because of the Rayleigh-Ritz principle. In order to show that (14) is bounded, we show that the following linear program is bounded from above:

15 $$\begin{aligned} \max _{y\in \mathbbm {R}^I_{\ge 0}} \varepsilon ^\top y:\ \tau ^\top y \le \sum _{i=1}^k x_i \mathbbm {1}_{X_i}^c(\mu _{}), \end{aligned}$$

where $\tau _i = \text {sign}(\varepsilon _i) \mathbbm {1}_{T_i}(\mu _{}).$ Note that $\tau \ne 0$ due to $\mu _{} \in T_2$. Similar to before, the constraint in (15) can be derived from (11b) with $t=\mu _{}$ in the following way:

16 $$\begin{aligned} \begin{aligned} \sum _{i=1}^k x_i \mathbbm {1}_{X_i}^c(\mu _{})&\ge \sum _{i=1}^k x_i \mathbbm {1}_{X_i}^c(\mu _{}) - \langle \begin{bmatrix} \varSigma &  0 \\ 0 &  \varepsilon _{\mu _{}} \end{bmatrix}, Y_1 \rangle \ge \sum _{i\in I} \text {sign}(\varepsilon _i) \mathbbm {1}_{T_i}^c(\mu _{})y_i. \end{aligned} \end{aligned}$$

Then, weak duality implies

$$\begin{aligned} (15) \le \mathop {\textrm{min}}\limits _{z\in \mathbbm {R}_{\ge 0}} z \sum _{i=1}^k x_i \mathbbm {1}_{X_i}^c(\mu _{}):\ z \tau -\varepsilon \ge 0. \end{aligned}$$

Observe that $z=1$ is a feasible solution since

$$\tau _i=\text {sign}(\varepsilon _i)\mathbbm {1}_{T_i}^c(\mu  )=1>\varepsilon _i$$

for every $i\in I\setminus \{1\}$ and $\tau _1=-1=\varepsilon _1$. Thus, we obtain an upper bound for (15) and thereby for $\text {Tr}(Y_2)$. Finally, we proved that the coefficients of $p_Y(t)$ are bounded, and the claim follows. $\square$

Observe that the assumptions on the confidence sets $T_i$, i.e., that either it is $\mu _{} \in T_i$ whenever $\varepsilon _i>0$ or $\mu _{} \notin T_i$ if $\varepsilon _i<0$, limits the power of modeling ambiguity sets $\mathcal {P}$. Indeed, we our model does not include upper bounds on $\mathbbm {P}(T_i)$ if $\mu \in T_i$ and lower bounds if $\mu \notin T_i$. We note that this limitation is rather mild, as most real-world distributions are concentrated around their respective expectations to some degree. Consequently, since the requirement above still allows us to force the probability mass of $\mathbbm {P}_{}\in \mathcal {P}$ towards the estimated expected value $\mu _{}$, it seems not very restrictive in practice. In fact, discrepancy-based approaches such as Wasserstein balls yield a similar structure.

If confidence sets are used, restrictions in modeling are fairly common, also, for example, in the so-called nesting condition in [40] and the references therein. In addition, there are relevant settings where the assumption from the above lemma can be weakened. Indeed, in [16] it is shown that for one-dimensional *T*, no such assumption is needed at all.

In the following lemma, we establish an inner approximation of the DRO constraint (11b). To this end, we denote by $T_N=\delta _N \mathbbm {Z}^m\cap T$ the standard lattice with stepsize $\delta _N \in \mathbbm {R}_{>0}$, that serves as a discretization of *T*. Moreover, we define a *level set* $L_h$ by

$$L_h:=\left\{ t\in T:\ \sum _{i=1}^k x_i \mathbbm {1}_{X_i}(t)-\sum _{i\in I} \text {sign}(\varepsilon _i) \mathbbm {1}_{T_i}(t) =h\right\} ,$$

where *h* denotes the *height* of the specific level set. The motivation to consider these level sets is that on the boundaries of $L_h$ the indicator functions $\mathbbm {1}_{X_i},\mathbbm {1}_{T_i}$ abruptly change and any potential Lipschitz constant *L* for the continuous approximations $\mathbbm {1}_{X_i}^c,\mathbbm {1}_{T_i}^c$ of $\mathbbm {1}_{X_i},\mathbbm {1}_{T_i}$ tends to infinity the closer the continuous approximation is. Consequently, an approximation of the left-hand side of (11b) solely based on Lipschitz continuity may become quite poor.

Thus, we address the indicator functions separately. To this end, let us first denote

$$\begin{aligned} f^{c}(t):=&\sum _{i=1}^k x_i \mathbbm {1}_{X_i}^{c}(t) - \left\langle \begin{bmatrix} \varSigma &  t-\mu _{}\\ (t-\mu _{})^\top &  \varepsilon _{\mu _{}} \end{bmatrix} , Y_1 \right\rangle +\langle (t-\mu _{})(t-\mu _{})^\top , Y_2\rangle \\&\quad -\sum _{i\in I} \text {sign}(\varepsilon _i) \mathbbm {1}_{T_i}^{c}(t) y_i \end{aligned}$$

for fixed $Y_1 \in \mathcal {S}^{m+1}_{\succeq 0}, Y_2 \in \mathcal {S}^m_{\succeq 0}, y \in \mathbbm {R}_{\ge 0}^I$ and observe the equivalence

$$(11b) \Leftrightarrow f^c(t)\ge 0 \text { for every } t \in T.$$

Let us further observe that in most applications, we can assume that $X_i\cap T_N\ne \emptyset$ and $T_i\cap T_N\ne \emptyset$, whenever $\delta _N$ is sufficiently small, e.g., if every $X_i$ and $T_i$ contains open sets. In particular, we assume that $\delta _N$ is chosen small enough such that for every $t\in L_h$, we have that there is a $\bar{t}\in T_N\cap L_h$ with $\Vert t-\bar{t}\Vert \le \sqrt{m}\delta _N$. Since $T_N = \delta _N \mathbbm {Z}^m\cap T$, this guarantees that for every $t\in L_h$, there is a nearby sample point also contained in $L_h$. Consequently, as seen in Lemma 4.1, we can address the differences on $f^c$ evaluated on sample points $\bar{t}\in T_N$ compared to the nearby non-sample points $t\in T\setminus T_N$ by exploiting Lipschitz continuity on the polynomial part *p* of $f^c$. Finally, we observe that the union of all these level sets $\bigcup _{h} L_h=T$ is a finite, disjoint decomposition of *T* and thus, we have addressed all potential deviations of $f^c$ between values on $T\setminus T_N$ and $T_N$. To make these arguments precise:

#### Lemma 4.2

Let $L>0$ be the Lipschitz constant of $p_Y$. Let further $\delta _N$ be sufficiently small, such that for every $t\in T$ with w.l.o.g. $t\in L_h$, there exists a $\bar{t}\in T_N\cap L_h$ with $\Vert t-\bar{t}\Vert \le \delta _N\sqrt{m}$. Then, the finitely many constraints

17 $$\begin{aligned} f(\bar{t})-L\delta _N\sqrt{m} \ge 0 \text { for every } \bar{t}\in T_N \end{aligned}$$

imply the semi-infinite constraint

$$f^c(t) \ge 0 \text { for every } t\in T.$$

#### Proof

We first suppose w.l.o.g. that $t\in L_h$. Then, there exists a $\bar{t}\in L_h$ such that $\Vert t-\bar{t}\Vert \le \delta _N\sqrt{m}$ and hence

$$\begin{aligned} f^c(t)+L\delta _N\sqrt{m}&\ge f^c(t)+L\Vert t-\bar{t}\Vert \overset{(1)}{\ge }\ f^c(t) +|p_Y(\bar{t})-p_Y(t)|\\&\overset{(11a)}{\ge } \sum _{i=1}^k x_i \mathbbm {1}_{X_i}^c(t) - \sum _{i\in I} \text {sign}(\varepsilon _i) \mathbbm {1}_{T_i}^c(t) + p_Y(\bar{t}) \\&\overset{(2)}{\ge }\ \sum _{i=1}^k x_i \mathbbm {1}_{X_i}(t) - \sum _{i\in I} \text {sign}(\varepsilon _i) \mathbbm {1}_{T_i}(t) + p_Y(\bar{t}) = f(\bar{t})\, \end{aligned}$$

where (1) holds due to definition of *L* and (2) holds due to (10). $\square$

Note that Lemma 4.2 provides a sufficient criterion for the SIP constraint (11b). Thus, replacing (11b) by (17) gives an inner approximation of (11). Therefore, the existence of $y,Y_1,Y_2$ satisfying (17) in addition to the remaining constraints of (11) guarantees that the DRO constraint (9) is satisfied.

### Tractable Approximations for DRO

We note that (9) is often considered as the (nonconvex) DRO constraint embedded in an otherwise convex program, e.g., as illustrated by Cases 1 and 2 in Section 3. Hence, instead of considering constant $x_i,X_i$, we investigate in the following paragraphs how the Lemma 4.2 approximation can be applied to Case 1, i.e., decision variables $x_i$ and Case 2, with decision variables $x_i^-,x_i^+$ that define the box $X_i=[x_i^-,x_i^+]$. For the sake of simplicity, we assume that the objective of DRO is linear. However, the results below hold analogously for maximizing concave objective functions as well. For Case 1 let $x\in C \subseteq \mathbbm {R}^n$ be a decision variable. We recall that $n=k$ and consider:

18a $$\begin{aligned} \max _{x, Y_1,Y_2,y}&c^\top x \end{aligned}$$

18b $$\begin{aligned} \text { s.t.}~&\sum _{i\in I} \varepsilon _i y_i -\varepsilon _\varSigma \langle \varSigma , Y_2 \rangle \ge b \end{aligned}$$

18c $$\begin{aligned}&\sum _{i=1}^k x_i \mathbbm {1}_{X_i}^c(t) - \left\langle \begin{bmatrix} \varSigma &  t-\mu _{}\\ (t-\mu _{})^\top &  \varepsilon _1 \end{bmatrix} , Y_1 \right\rangle \nonumber \\&+\left\langle (t-\mu _{})(t-\mu _{})^\top , Y_2\right\rangle -\sum _{i\in I} \text {sign}(\varepsilon _i) \mathbbm {1}_{T_i}^c(t) y_i\ge 0 \qquad \forall t\in T \end{aligned}$$

18d $$\begin{aligned}&x\in C, Y_1 \in \mathcal {S}^{m+1}_{\succeq 0}, Y_2 \in \mathcal {S}^m_{\succeq 0}, y \in \mathbbm {R}_{\ge 0}^I. \end{aligned}$$

It turns out that computing lower bounds for (18) is tractable:

#### Theorem 4.2

A solution to the following semidefinite problem yields a feasible solution to the semi-infinite problem (18).

19a $$\begin{aligned} \max _{x,Y_1,Y_2,y}\;&c^\top x \end{aligned}$$

19b $$\begin{aligned} \text { s.t.}~&\sum _{i\in I} \varepsilon _i y_i -\varepsilon _\varSigma \langle \varSigma , Y_2 \rangle \ge b \end{aligned}$$

19c $$\begin{aligned}&\sum _{i=1}^k x_i \mathbbm {1}_{X_i}(\bar{t}) - \left\langle \begin{bmatrix} \varSigma &  \bar{t}-\mu _{}\\ (\bar{t}-\mu _{})^\top &  \varepsilon _1 \end{bmatrix} , Y_1 \right\rangle +\langle (\bar{t}-\mu _{})(\bar{t}-\mu _{})^\top , Y_2\rangle \nonumber \\&-\sum _{i\in I} \text {sign}(\varepsilon _i) \mathbbm {1}_{T_i}(\bar{t}) y_i - L\delta _N\sqrt{m}\ge 0 \qquad \qquad \qquad \qquad \qquad \forall \bar{t}\in T_N \end{aligned}$$

19d $$\begin{aligned}&x\in C, Y_1 \in \mathcal {S}^{m+1}_{\succeq 0}, Y_2 \in \mathcal {S}^m_{\succeq 0}, y \in \mathbbm {R}_{\ge 0}^I. \end{aligned}$$

#### Proof

Given an arbitrary $x\in C$. Due to Lemma 4.2, we observe that Constraint (19c) implies $f^c(t)\ge 0$ for every $t\in T$, i.e. (18c). Hence, the claim follows. $\square$

We note that $\sum _{i=1}^k x_i \mathbbm {1}_{X_i}$ is linear and thus convex in the $x_i$. Thus, if the number of confidence sets |*I*| is low, Problem (19) satisfies the (weakened) conditions needed for Theorem 1 in [40] and can be exactly reformulated as a convex program by applying their methods, whereas the proposed method in this paper only provides a lower bound on (18). However, our approach can also be used for a large number of confidence sets. In addition, it does not depend on convexity and can also be used in nonconvex settings. This can be seen by the following result for Case 2, where $T=[0,M]^m$ and $X_i=[x_i^-,x_i^+]$ are supposed to be $k=2n$ hypercubes:

20a $$\begin{aligned} \max&\sum _{i=1}^k (c^-_i)^\top x_i^- + (c^+_i)^\top x_i^+ \end{aligned}$$

20b $$\begin{aligned} \text {s.t.}~&\sum _{i\in I} \varepsilon _i y_i -\varepsilon _\varSigma \langle \varSigma , Y_2 \rangle \ge b \end{aligned}$$

20c $$\begin{aligned}&\sum _{i=1}^k x_i \mathbbm {1}_{[x_i^-,x_i^+]}^c(t) - \left\langle \begin{bmatrix} \varSigma &  t-\mu _{}\\ (t-\mu _{})^\top &  \varepsilon _1 \end{bmatrix} , Y_1 \right\rangle \nonumber \\&\qquad +\left\langle (t-\mu _{})(t-\mu _{})^\top , Y_2\right\rangle -\sum _{i\in I} \text {sign}(\varepsilon _i) \mathbbm {1}_{T_i}^c(t) y_i\ge 0  &   \forall t\in T \end{aligned}$$

20d $$\begin{aligned}&x_i^-,x_i^+\in C, Y_1 \in \mathcal {S}^{m+1}_{\succeq 0}, Y_2 \in \mathcal {S}^m_{\succeq 0}, y \in \mathbbm {R}_{\ge 0}^I. \end{aligned}$$

We note that $\sum _{i=1}^k x_i \mathbbm {1}_{[x_i^-,x_i^+]}^c$ is nonconvex in the variables $x_i^-,x_i^+\in \mathbbm {R}^m$. In the following theorem, we model the indicator function $\mathbbm {1}_{[x_i^-,x_i^+]}^c:T_N\rightarrow \mathbbm {R}$ by binary variables $\tilde{b}_{\bar{t}}^i$. Additionally, we ensure that these variables properly model $\mathbbm {1}_{[x_i^-,x_i^+]}^c(\bar{t})$ by tracking the "jumps" from 0 to 1 at $x_{ij}^-$ in direction $j\in [m]$ by additional binary variables $\varDelta _{\bar{t}}^{-,i,j}$ and the "jumps" form 1 to 0 at $x_{ij}^+$ in direction $j\in [m]$ by $\varDelta _{\bar{t}}^{+,i,j}$ respectively. For univariate simple functions, a modeling approach along these lines was given in [15] for an engineering application in the design of particulate products.

#### Theorem 4.3

Let $M_\delta :=\{0,\delta _N,\ldots , M\}$ the discretization of [0, *M*], $T_0^j=\{\bar{t}\in T_N: \bar{t}_j=0\}\subseteq T_N$ a set of boundary points of $T_N = \delta _N\mathbbm {Z}^m\cap [0,M]^m$. Then, a solution to the following MISDP yields a feasible solution to (20).

21a $$\begin{aligned} \max&\sum _{i=1}^k (c^-_i)^\top x_i^- + (c^+_i)^\top x_i^+ \end{aligned}$$

21b $$\begin{aligned} \text {s.t.}~&\sum _{i\in I} \varepsilon _i y_i -\varepsilon _\varSigma \langle \varSigma , Y_2 \rangle \ge b \end{aligned}$$

21c $$\begin{aligned}&\sum _{i=1}^k x_i\tilde{b}_{\bar{t}}^i - \left\langle \begin{bmatrix} \varSigma &  \bar{t}-\mu _{}\\ (\bar{t}-\mu _{})^\top &  \varepsilon _1 \end{bmatrix} , Y_1 \right\rangle \nonumber \\&\qquad +\langle (\bar{t}-\mu _{})(\bar{t}-\mu _{})^\top , Y_2\rangle \nonumber \\&\qquad -\sum _{i\in I} \text {sign}(\varepsilon _i) \mathbbm {1}_{T_i}(\bar{t}) y_i -L\delta _N\sqrt{m}\ge 0  &   \forall \bar{t}\in T_N \end{aligned}$$

21d $$\begin{aligned}&\tilde{b}_{\bar{t} + e_j\delta _N}^i -\tilde{b}_{\bar{t}}^i = \varDelta _{\bar{t}}^{-,i,j}-\varDelta _{\bar{t}}^{+,i,j}  &   \forall \bar{t}\in T_N, i\in [k], j\in [m] \end{aligned}$$

21e $$\begin{aligned}&\sum _{\begin{array}{c} l\in M_\delta :\\ \bar{t}=t_0+le_j \end{array}} \varDelta _{\bar{t}}^{-,i,j}+\varDelta _{\bar{t}}^{+,i,j} \le 2  &   \forall i\in [k], j\in [m], t_0\in T_0^j \end{aligned}$$

21f $$\begin{aligned}&x_{ij}^-\ge \sum _{\begin{array}{c} l\in M_\delta :\\ \bar{t}=t_0+le_j \end{array}} (l+\delta _N) \varDelta _{\bar{t}}^{-,i,j}  &   \forall i\in [k],j\in [m], t_0\in T_0^j \end{aligned}$$

21g $$\begin{aligned}&x_{ij}^+\le M-\sum _{\begin{array}{c} l\in M_\delta :\\ \bar{t}=t_0+le_j \end{array}} (M-l) \varDelta _{\bar{t}}^{+,i,j}  &   \forall i\in [k], j\in [m], t_0\in T_0^j \end{aligned}$$

21h $$\begin{aligned}&x_{ij}^+-x_{ij}^- \ge M \sum _{\begin{array}{c} l\in M_\delta :\\ \bar{t}=t_0+le_j \end{array}} \varDelta _{\bar{t}}^{+,i,j}\nonumber \\&\quad \ -\hspace{-0.38cm}\sum _{\begin{array}{c} l\in M_\delta :\\ \bar{t}=t_0+le_j \end{array}} \left( (M-l) \varDelta _{\bar{t}}^{+,i,j} - (l+\delta _N) \varDelta _{\bar{t}}^{-,i,j}\right)  &   \forall i\in [k], j\in [m], t_0\in T_0^j \end{aligned}$$

21i $$\begin{aligned}&0 \le x_{ij}^+ -x_{ij}^-\le \delta _N (\sum _{\begin{array}{c} l\in M_\delta :\\ \bar{t}=t_0+le_j \end{array}} \tilde{b}_{\bar{t}}^i-1)  &   \forall i\in [k],\forall j\in [m], t_0\in T_0^j \end{aligned}$$

21j $$\begin{aligned}&x_i^-,x_i^+\in C, y \in \mathbbm {R}_{\ge 0}^I, Y_1 \in \mathcal {S}^{m+1}_{\succeq 0}, Y_2 \in \mathcal {S}^m_{\succeq 0} \end{aligned}$$

21k $$\begin{aligned}&\varDelta _{\bar{t}}^{-,i,j},\varDelta _{\bar{t}}^{+,i,j},\tilde{b}_{\bar{t}}^i\in \{0,1\}, \end{aligned}$$

where $\tilde{b}_{\bar{t}}^i:=0$ for every $\bar{t}\notin T_N$.

We would like to point out that we could also extend this model further. Indeed, instead of fixed $x_i$ in Theorem 4.3, we could additionally include $x_i$ as a bounded decision variable. This is due to the fact that for bounded $x_i$ the arising bilinear term $x_i\tilde{b}_{\bar{t}}^i$ in Constraint (21c) can be rewritten as a linear term with the help of additional big-M constraints.

#### Proof

We consider a feasible solution $\varDelta _{\bar{t}}^{-,i,j},\varDelta _{\bar{t}}^{+,i,j},\tilde{b}_{\bar{t}}^i, x_i^-,x_i^+$ for (21) and show that for every $i\in [k], \bar{t}\in T_N$ we have $\tilde{b}_{\bar{t}}^i=\mathbbm {1}_{[x_i^-,x_i^+]}(\bar{t})$. To this end, note that for every $i\in [k]$ there exists indeed an index $\bar{t}$ with $\tilde{b}_{\bar{t}}^i=1$ due to (21i). Now, given an arbitrary index $\bar{t}$ with $\tilde{b}_{\bar{t}}^i=1$, we first show that $\tilde{b}_{\bar{t}}^i=1$ implies $\mathbbm {1}_{[x_i^-,x_i^+]}(\bar{t})=1$, i.e., $\bar{t}\in [x_i^-,x_i^+]$:

We first observe that for every direction *j*, there exists a $t_0\in T_0^j$ and $\kappa _j\in \{0,\delta _N,2\delta _N,\ldots ,M\}$ such that

$$\bar{t} = t_0+\kappa _j e_j,$$

i.e., we consider the line in direction *j* passing through $\bar{t}$ and consequently through $t_0$ as well. Then, we define $\kappa _j^{\max }$ as the index of the last element on this line with $\tilde{b}_t^i=1$, i.e.,

$$\kappa _j^{\max }:=\max \{ l\in \{0,\delta _N,2\delta _N,\ldots ,M\}: \tilde{b}_{t_0+le_j}^i=1\}.$$

Thus, $\tilde{b}_{t_0+(\kappa _j^{\max }+\delta _N)e_j}^i=0$ and (21d) implies $\varDelta _{t_0+\kappa _j^{\max }e_j}^{-,i,j}=0, \varDelta _{t_0+\kappa _j^{\max }e_j}^{+,i,j}=1$. Moreover, (21g) implies

22 $$\begin{aligned} x_{ij}^+ \le M-(M-\kappa _j^{\max })=\kappa _j^{\max }=\bar{t}_j + (\kappa _j^{\max }-\kappa _j), \end{aligned}$$

where the latter equality originates from the definition of $\kappa _j$ above. Similarly, we define

$$\kappa _j^{\mathop {\textrm{min}}\limits }:=\mathop {\textrm{min}}\limits \{l\in \{0,\delta _N,2\delta _N,\ldots ,M\}: \tilde{b}_{t_0+le_j}^i=1\}.$$

Thus, $\tilde{b}_{t_0+(\kappa _j^{\mathop {\textrm{min}}\limits }-\delta _N)e_j}^i=0$ and (21d) implies $\varDelta _{t_0+(\kappa _j^{\mathop {\textrm{min}}\limits }-\delta _N)e_j}^{-,i,j}=1, \varDelta _{t_0+(\kappa _j^{\mathop {\textrm{min}}\limits }-\delta _N)e_j}^{+,i,j}=0$. Moreover, (21f) implies

23 $$\begin{aligned} x_{ij}^- \ge (\kappa _j^{\mathop {\textrm{min}}\limits }-\delta _N)+\delta _N=\kappa _j^{\mathop {\textrm{min}}\limits } = \bar{t}_j + \kappa _j^{\mathop {\textrm{min}}\limits }-\kappa _j. \end{aligned}$$

However, due to (21e) we know that these are the only nonzero entries for $\varDelta _{t_0+le_j}^{-,i,j},\varDelta _{t_0+le_j}^{+,i,j}$. Thus due to (21h), we obtain

$$x_{ij}^+-x_{ij}^- \ge M - (M-\kappa _j^{\max })-\kappa _j^{\mathop {\textrm{min}}\limits } = \kappa _j^{\max }-\kappa _j^{\mathop {\textrm{min}}\limits },$$

which implies equality in both (23) and (23) and thus $\bar{t}_j=\kappa _j\in [\kappa _j^{\mathop {\textrm{min}}\limits },\kappa _j^{\max }]=[x_{ij}^-, x_{ij}^+]$ for every index $\bar{t}\in T_N$ with $\tilde{b}_{\bar{t}}^i=1$.

For the reverse implication, we need to show that $\bar{t}\in [x_i^-,x_i^+]$ implies $\tilde{b}_{\bar{t}}^i=1$. Due to (21i), we obtain that $[x_i^-,x_i^+]\ne \emptyset$ implies the existence of a $\bar{t}$ with $\tilde{b}_{\bar{t}}^i=1$. In particular, the previous implication shows that $\bar{t}\in [x_i^-,x_i^+]$. Beginning with this $\bar{t}$, we prove the following claim for an arbitrary direction *j*:

24 $$\begin{aligned} \tilde{b}_{\bar{t}}^i=1 \text { implies } \tilde{b}_{\bar{t}+le_j}^i =1 \text { for every } l: \bar{t}_j+l\in [x_{ij}^-, x_{ij}^+]. \end{aligned}$$

Let $\bar{t}=t_0+\kappa _je_j$ with $t_0\in T_0^j$ as above. Then, with the same definitions for $\kappa _j^{\mathop {\textrm{min}}\limits },\kappa _j^{\max }$, the arguments from the previous implication that led to equality in (23) and (23) imply $\kappa _j^{\mathop {\textrm{min}}\limits }=x_{ij}^-$, $\kappa _j^{\max }=x_{ij}^+$. Moreover, the definition of $\kappa _j^{\mathop {\textrm{min}}\limits }, \kappa _j^{\max }$ leads to:

$$1=\tilde{b}_{t_0+\kappa _j^{\mathop {\textrm{min}}\limits }e_j}^i=\tilde{b}_{t_0+(\kappa _j^{\mathop {\textrm{min}}\limits }+\delta _N)e_j}^i=\ldots =\tilde{b}_{t_0+\kappa _j^{\max }e_j}^i=1$$

with $(t_0+\kappa _j^{\mathop {\textrm{min}}\limits }e_j)_j=x_{ij}^-$ and $(t_0+\kappa _j^{\max }e_j)_j=x_{ij}^+$. Hence, our claim (24) follows and as the direction *j* was chosen arbitrarily, we obtain that $\mathbbm {1}_{[x_i^-,x_i^+]}(\bar{t})=1$ also implies $\tilde{b}_{\bar{t}}^i=1$. $\square$

Theorem 4.3 yields a sufficient criterion for the DRO constraint to be satisfied. This is a considerable advantage, as to our knowledge, no practically efficient alternative approach is readily available. Positive semidefinite optimization is algorithmically tractable, and recent research has been successful in enhancing global solution algorithms when binary variables are present as well. Nevertheless, solving a binary SDP is still more elaborate than solving binary linear optimization models. As a result, (21) may be computationally too involved even for modern solvers for a large cardinality of $T_N$. For one-dimensional domains *T* as considered in [16], this challenge has been addressed as follows: Instead of bounding the slope of $p_Y$ through its Lipschitz constant *L*, more elaborate bounds that strengthen Lemma 4.2 reduce the number of necessary sample points for a good approximation of (20). Moreover, due to the one-dimensional domain *T*, instead of a binary SDP, we obtain a binary MIP as an approximation of (20) that can typically be solved much faster in practice. We next show some preliminary computational results for the SDP model presented here.

## Computational Results

In this section, we show some preliminary computational results for solving model (21) via available binary SDP solvers. We restrict ourselves to solving an illustrative toy example that is easily comprehensible.

### Example 5.1

(Bin creating problem) Given an *m*-dimensional random variable $t\in T=[0,M]^m$, where focus on $m=2, M=1$ here. Let it be distributed according to a distribution that is contained in a set of probability distributions $\mathcal {P}$. We suppose further that the best-known estimates for the expectation of *t* is $\mu =(0,0)^\top$ and $$\varSigma =\begin{pmatrix} 2 &  0.5 \\ 0.5 &  1 \end{pmatrix}$$. Then, we ask for a representative box $[x^-,x^+]\in \mathbbm {R}^2$ for the ambiguity set $\mathcal {P}$ as follows:

25a $$\begin{aligned} \mathop {\textrm{min}}\limits _{x_1^-,x_1^+,x_2^-,x_2^+}&|x_1^+-x_1^-| + |x_2^+-x_2^-| \end{aligned}$$

25b $$\begin{aligned} \text { s.t.}~&0.1 \le \mathop {\textrm{min}}\limits _{\mathbbm {P}\in \mathcal {P}} \mathbbm {P}([x_i^-,x_i^+])  &   \forall i\in \{1,2\}, \end{aligned}$$

25c $$\begin{aligned}&x_i^+ - x_i^- \ge 0  &   \forall i\in \{1,2\},\end{aligned}$$

25d $$\begin{aligned}&x_1^-,x_1^+,x_2^-,x_2^+\in \mathbbm {R}_{\ge 0}, \end{aligned}$$

where, due to $\varepsilon _\mu =0.1, \varepsilon _\varSigma =1$, we set

$$\mathcal {P}=\{\mathbbm {P}\in \mathcal {M}(T)_{\ge 0}: \mathbbm {P}(T)=1, (\mathbbm {E}_{\mathbbm {P}}(t)-\mu )^\top \varSigma (\mathbbm {E}_{\mathbbm {P}}(t)-\mu ) \le 0.1, \text {Var}(t) \preceq \varSigma \}.$$

Since our primary interest lies in the characteristic behavior of the approach, we refrain from introducing additional constraints into the model.

We note that (25a) can be linearized by adding auxiliary variables $z_1,z_2\in \mathbbm {R}_{\ge 0}$, the additional constraints $z_i \ge \pm (x_i^+ - x_i^-)$ and by replacing the objective by $z_1 + z_2$. Moreover, to specify the continuous counterpart of the indicator functions in a manner to satisfy the key property (10), we set

$$\mathbbm {1}_{[x_i^-,x_i^+]}^c(s) = \frac{d_H(\{s\},(-\infty ,x_i^--\delta _N] \cup [x_i^+ + \delta _N,\infty ))}{d_H(\{s\},[x_i^-,x_i^+]) + d_H(\{s\},(-\infty ,x_i^--\delta _N] \cup [x_i^+ + \delta _N,\infty ))},$$

where $d_H$ denotes the *Hausdorff distance* on $\mathbbm {R}$, i.e.,

$$d_H(X,Y):=\max \left\{ \sup _{x\in X}\inf _{y\in Y} |x-y|,\sup _{y\in Y}\inf _{x\in X} |x-y| \right\} .$$

We note that $\mathbbm {1}_{[x_i^-,x_i^+]}^c(s) \ge \mathbbm {1}_{[x_i^-,x_i^+]}(s)$ since $\mathbbm {1}_{[x_i^-,x_i^+]}^c(s)\ge 0$ and for $s\in [x_i^-,x_i^+]$, we have that $\mathbbm {1}_{[x_i^-,x_i^+]}^c(s)=1$. The continuity is given by the Lemma of Urysohn; see e.g. [30].

As the univariate functions in the maximum term are both nonnegative, the above inequality holds for their respective product as well, and we obtain

$$\begin{aligned} \mathbbm {1}_{[x^-,x^+]}^c(t)&:=\prod _{i=1}^m \mathbbm {1}_{[x_i^-,x_i^+]}^c(t_i) \ge \prod _{i=1}^m \mathbbm {1}_{[x_i^-,x_i^+]}(t_i) = \mathbbm {1}_{[x^-,x^+]}(t). \end{aligned}$$

The indicator function $\mathbbm {1}_{T_i}$ is the constant function having value one and thus continuous for $T_i=T$. Therefore, (10) is satisfied.

Hence, the only parameter not yet determined is the Lipschitz constant *L* of the polynomial $$p_Y(t)=\left\langle \begin{bmatrix} \varSigma &  t-\mu _{}\\ (t-\mu _{})^\top &  \varepsilon _{\mu _{}} \end{bmatrix}, Y_1 \right\rangle +\langle (t-\mu _{})(t-\mu _{})^\top , Y_2\rangle .$$ However, if we follow the proof in Lemma 4.1, we obtain an upper bound of

$$\begin{aligned} \lambda _{\mathop {\textrm{min}}\limits }\left( \begin{bmatrix} \varSigma &  0 \\ 0 &  \varepsilon _{\mu _{}} \end{bmatrix}\right) \text {Tr}(Y_1)\le &   \sum _{i=1}^k x_i \mathbbm {1}_{X_i}^c(\mu _{}) - \sum _{i\in I} \text {sign}(\varepsilon _i) y_i \le k + y_1 - y_2 \\\le &   1 + b =1.1 \end{aligned}$$

due to (12). Hence, with $$\lambda _{\mathop {\textrm{min}}\limits }\left( \begin{bmatrix} 2 &  0.5 &  0\\ 0.5 &  1 &  0\\ 0 &  0 &  1\end{bmatrix}\right) = \frac{3-\sqrt{2}}{2} \approx 0.8$$, we obtain $\text {Tr}(Y_1) \le 1.4$. Similarly, we obtain an upper bound of

$$\begin{aligned} \varepsilon _\varSigma \lambda _{\mathop {\textrm{min}}\limits }(\varSigma ) \text {Tr}(Y_2) \le \sum _{i\in I} \varepsilon _i y_i = -y_1 + y_2 \le 1 \end{aligned}$$

due to (15). We have also invested that $\varepsilon = \tau = (-1,1)^\top$. Hence, with $\lambda _{\mathop {\textrm{min}}\limits }(\varSigma ) \approx 0.8$, we obtain $\text {Tr}(Y_2) \le 1.3$.

With these bounds it is now possible to determine a Lipschitz constant for $p_Y$. Please note that the following proposition holds for general values of *m* and can be combined with the above bounds

$$\text {Tr}(Y_1) \le \frac{1+b}{ \lambda _{\mathop {\textrm{min}}\limits }\left( \begin{bmatrix} \varSigma &  0 \\ 0 &  \varepsilon _{\mu  } \end{bmatrix}\right) } \text { and } \text {Tr}(Y_2) \le \frac{1}{\varepsilon _\varSigma \lambda _{\mathop {\textrm{min}}\limits }(\varSigma )},$$

if $\varSigma \succ 0, \varepsilon _{\mu _{}},\varepsilon _\varSigma >0$ in order to determine a potential Lipschitz constant for (21c).

### Proposition 1

Let $t\in T=[0,M]^m$, $\mu _{\mathop {\textrm{min}}\limits }:=\mathop {\textrm{min}}\limits _{i\in [m]}\{\mu _i\}$ and assume that $\mu _{\mathop {\textrm{min}}\limits } \le \frac{M}{2}$. Then, the polynomial is Lipschitz continuous on the compact set *T* with Lipschitz constant

$$L=2\text {Tr}(Y_1) + (M-\mu _{\mathop {\textrm{min}}\limits })\text {Tr}(Y_2)2\sqrt{m}.$$

### Proof

We first observe that

$$p_Y(t) - p_Y(t') = \left\langle \begin{bmatrix} 0 &  t-t'\\ (t-t')^\top &  0 \end{bmatrix}, Y_1 \right\rangle +\langle (t-\mu  )(t-\mu  )^\top - (t'-\mu  )(t'-\mu  )^\top , Y_2\rangle .$$

We continue by bounding the terms separately. For the first term we obtain:

$$\begin{aligned} \left\langle \begin{bmatrix} 0 &  t-t'\\ (t-t')^\top &  0 \end{bmatrix} , Y_1 \right\rangle&\le \Vert \begin{bmatrix} 0 &  t-t'\\ (t-t')^\top &  0 \end{bmatrix}\Vert _F \Vert Y_1\Vert _F\\&= \sqrt{2\Vert t-t'\Vert _2^2} \sqrt{\text {Tr}(Y_1^\top Y_1)}\\&= \sqrt{2} \Vert t-t'\Vert _2 \sqrt{\sum _{l=1}^{m+1} \lambda _l^2} \le \sqrt{2} \Vert t-t'\Vert _2 \text {Tr}(Y_1), \end{aligned}$$

where the first inequality is due to Cauchy-Schwartz’ inequality and the last step is due to the equivalence of norms. Similarly,

$$\begin{aligned}&\langle (t-\mu _{})(t-\mu _{})^\top - (t'-\mu _{})(t'-\mu _{})^\top , Y_2\rangle \\  &\le \sum _{i,j\in [m]} [(t_i-\mu _i)(t_j-\mu _j) - (t_i'-\mu _i)(t_j'-\mu _j)]\\&= \text {Tr}(Y_2) \sqrt{\sum _{i,j\in [m]} [t_it_j - t_i\mu _j -\mu _it_j - t_i't_j' + t_i'\mu _j + \mu _it_j']^2}\\&= \text {Tr}(Y_2) \sqrt{\sum _{i,j\in [m]} [t_it_j - t_i't_j' - (t_i-t_i')\mu _j -\mu _i (t_j-t_j')]^2}\\&= \text {Tr}(Y_2) \sqrt{\sum _{i,j\in [m]} [(t_i-t_i')t_j + t_i'(t_j-t_j') - (t_i-t_i')\mu _j -\mu _i (t_j-t_j')]^2}\\&= \text {Tr}(Y_2) \sqrt{\sum _{i,j\in [m]} [(t_j-\mu _j) (t_i-t_i') + (t_i'-\mu _i)(t_j-t_j')]^2}\\&\le \text {Tr}(Y_2)(M-\mu _{\mathop {\textrm{min}}\limits } ) \sqrt{ \sum _{i,j\in [m]} [(t_i-t_i') + (t_j-t_j')]^2}\\&= \text {Tr}(Y_2)(M-\mu _{\mathop {\textrm{min}}\limits } ) 2\sqrt{m} \Vert t-t'\Vert _2, \end{aligned}$$

where we utilize Cauchy-Schwartz for the first inequality as above and apply the assumption $\mu _{\mathop {\textrm{min}}\limits } \le \frac{M}{2}$. $\square$

We note that for random vectors where the assumption $\mu _{\mathop {\textrm{min}}\limits } \le \frac{M}{2}$ is violated, one may also be able to represent Problem (5.1) appropriately by choosing the domain $[0,M]^m +\mu _{\mathop {\textrm{min}}\limits }$.

With these notations, the model (21) could now be solved by an appropriate binary SDP solver. For this specific toy example, however, the problem structure can be exploited further to lead to a smaller model. Namely, we simplify the safe approximation (21) to the following model

26a $$\begin{aligned} \mathop {\textrm{min}}\limits \  &x^+_1-x^-_1 + x^+_2-x^-_2 \end{aligned}$$

26b $$\begin{aligned} \text {s.t.}~\  &-y_1+y_2 -\varepsilon _\varSigma \langle \varSigma , Y_2 \rangle \ge b \end{aligned}$$

26c $$\begin{aligned}&\tilde{b}_{\bar{t}} - \left\langle \begin{bmatrix} \varSigma &  \bar{t}-\mu _{}\\ (\bar{t}-\mu _{})^\top &  \varepsilon _1 \end{bmatrix} , Y_1 \right\rangle \nonumber \\&\qquad +\langle (\bar{t}-\mu _{})(\bar{t}-\mu _{})^\top , Y_2\rangle \nonumber \\&\qquad -\sum _{i\in I} \text {sign}(\varepsilon _i) \mathbbm {1}_{T_i}(\bar{t}) y_i -L\delta _N\sqrt{m}\ge 0  &   \forall \bar{t}\in T_N \end{aligned}$$

26d $$\begin{aligned}&x_{j}^+ - x_{j}^- \ge \delta _N (\sum _{\begin{array}{c} l\in M_\delta :\\ \bar{t}=t_0+le_j \end{array}} \tilde{b}_{\bar{t}}^i-1)  &   \forall i\in [k],\forall j\in [m], \forall t_0\in T_0^j \end{aligned}$$

26e $$\begin{aligned}&x^-,x^+\in [0,M]^m, y \in \mathbbm {R}_{\ge 0}^I, \nonumber \\&Y_1 \in \mathcal {S}^{m+1}_{\succeq 0}, Y_2 \in \mathcal {S}^m_{\succeq 0}, \end{aligned}$$

26f $$\begin{aligned}&\tilde{b}\in \{0,1\}^{T_N}. \end{aligned}$$

The problem formulation (26) strongly depends on the discretization width. On the one hand, if $\delta _N$ is too large, the safety term $L\delta _N\sqrt{m}$ in (26c) leads to an infeasible model. On the other hand, if $\delta _N$ is too small, the number of binary variables $\tilde{b}_{\bar{t}}$ in (26) grows polynomially in this width, for $m=2$ quadratically. Despite the considerable progress in solving binary SDPs in recent years, these models are still demanding for a large number of binary variables. We thus vary the discretization width in our experiments.

The computational experiments have been performed with the parameters $c,\varepsilon _i,\varepsilon _\mu , \varepsilon _\varSigma , \mu , \varSigma , L$ and *M* chosen as above. In order to solve the resulting instance of  (26), we utilized the state-of-the-art solver SCIP-SDP as presented in e.g., [18], where the SDP subproblems have been solved by MOSEK [29]. The computations have been executed on a MacBookAir 2024 with M3 chip and 16GB memory.

**Fig. 1:**
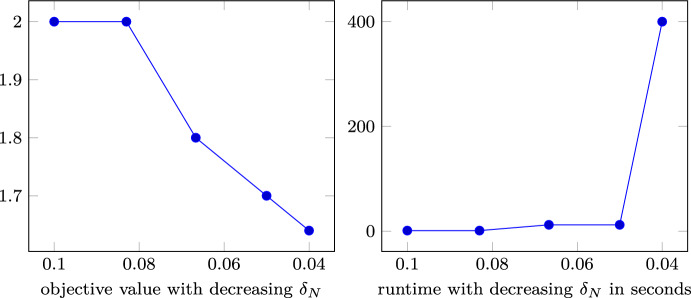
Development of objective values and runtime for $M=1$, $b=0.1$, $\varepsilon _\mu =0.1$, $\varepsilon _\varSigma =1$, $\mu =0$ and $$\varSigma =\begin{bmatrix} 2 &  0.5\\ 0.5 &  1 \end{bmatrix}$$

Figure 1 displays the objective function value of the objective as a function of discretization width $\delta _N$ (left) and the running time as a function of $\delta _N$ (right). Here, the choice of $\delta _N$ is crucial, as the number of binary variables in (26) is given by $\left( \frac{M}{\delta _N}\right) ^m$, here $\left( \frac{1}{\delta _N}\right) ^2$.

It is worth noting that the solution $x^-_i=-1, x^+_i=1, \tilde{b}=1, y_2=b$ and vanishing $Y_1,Y_2,y_i$ for $i\ne 2$ is feasible if $1-b-L\delta _N \sqrt{m} \ge 0$ and $\delta _N \le \frac{1-b}{L\sqrt{m}}$. Thus, choosing $\delta _N \le \frac{1-b}{L\sqrt{m}}$ guarantees a feasible safe approximation, but smaller values for $\delta _N$ reduce the safety term $L\delta _N\sqrt{m}$ and thereby enable a less conservative approximation of the original problem (5.1). From the left figure, we observe that this allows for a smaller box to capture the required probability mass of $10\%$.

However, as the number of binary variables increases at a rate of $\left( \frac{1}{\delta _N}\right) ^2$, the required running time quickly increases as well, as can be seen in the right figure. While model (26) can be solved to global optimality within a few seconds for a discretization width of up to 0.05, the required running time for $\delta _N=0.04$ is about 400 seconds. As the reduction in objective function value slows down for smaller discretization width, it can be assumed that a discretization of $\delta _N=0.04$ reasonably balances running time and quality of the safe approximation. We do not display smaller discretization widths here as the corresponding runtime exceeded a limit of 1h.

This section has served to illustrate the safe approximation via an illustrative example. It is evident that the model (21) as well as its variant (26) can be computationally demanding, in particular for small discretization widths. There are two future research directions to mitigate this: First, binary SDP solvers are expected to improve further over time, as this is an active area of research. Second, model (26) is a generic formulation designed to accommodate the general problem structure. To the best of our knowledge, it is the first such safe approximation for multivariate simple functions presented in the literature. By exploiting specific problem structures, it is expected that the formulation can be made smaller, thus leading to an improved solvability.

## Conclusions

In this paper, we present an extension of the novel approach in [16] for distributionally robust optimization problems to cases where multivariate simple functions are allowed. As simple functions can be included in the model, the presented approximation pushes the applicability of duality-based reformulations of distributional robustness significantly beyond convexity. Moreover, early convergence results from [16] for univariate indicator functions indicate that the presented approximation may converge to the actual optimum. A proof for this convergence as well as an extension from simple functions to more general functions is a desirable goal for future research.

With respect to algorithmic tractability, we have shown that a suitably discretized safe approximation yields a mixed-integer positive-semidefinite optimization model, making it eligible for recent MISDP approaches as presented in e.g., [18] or the YALMIP framework [27]. Thus, the presented formulation is tractable by using state-of-the-art solvers for MISDP, which we have also shown by computational results for an academic example. As running times strongly scale with the discretization width, future research will aim to downsize the model without incurring loss in obtained quality.

## Data Availability

No datasets were generated or analysed during the current study.
